# *N*‐acetylcysteine attenuates oxidative stress‐mediated cell viability loss induced by dimethyl sulfoxide in cryopreservation of human nucleus pulposus cells: A potential solution for mass production

**DOI:** 10.1002/jsp2.1223

**Published:** 2022-10-01

**Authors:** Shota Tamagawa, Daisuke Sakai, Jordy Schol, Kosuke Sako, Yoshihiko Nakamura, Erika Matsushita, Takayuki Warita, Soma Hazuki, Hidetoshi Nojiri, Masato Sato, Muneaki Ishijima, Masahiko Watanabe

**Affiliations:** ^1^ Department of Medicine for Orthopaedics and Motor Organ Juntendo University Graduate School of Medicine Tokyo Japan; ^2^ Department of Orthopaedic Surgery, Surgical Science Tokai University School of Medicine Isehara Japan; ^3^ Research Center for Regenerative Medicine Tokai University School of Medicine Isehara Japan; ^4^ TUNZ Pharma Co., Ltd. Osaka Japan

**Keywords:** cell therapy, cryopreservation, dimethyl sulfoxide, discogenic cell, intervertebral disc degeneration, N‐acetylcysteine, nucleus pulposus cells, oxidative stress, regenerative medicine

## Abstract

**Background:**

Cell therapy is considered a promising strategy for intervertebral disc (IVD) regeneration. However, cell products often require long‐term cryopreservation, which compromises cell viability and potency, thus potentially hindering commercialization and off‐the‐shelf availability. Dimethyl sulfoxide (DMSO) is a commonly used cryoprotectant, however, DMSO is associated with cytotoxicity and cell viability loss. This study aimed to investigate the effects of DMSO on human nucleus pulposus cells (NPC) and the role of oxidative stress in DMSO‐induced cytotoxicity. Furthermore, we examined the potential of antioxidant *N*‐acetylcysteine (NAC) supplementation to mitigate the negative effects of DMSO.

**Methods:**

NPC were exposed to various concentrations of DMSO with or without a freezing cycle. Cell viability, cell apoptosis and necrosis rates, intracellular reactive oxygen species (ROS) levels, and gene expression of major antioxidant enzymes were evaluated. In addition, NAC was added to cryopreservation medium containing 10% DMSO and its effects on ROS levels and cell viability were assessed.

**Results:**

DMSO concentrations ≤1% for 24 h did not significantly affect the NPC viability, whereas exposure to 5 and 10% DMSO (most commonly used concentration) caused cell viability loss (loss of 57% and 68% respectively after 24 h) and cell death in a dose‐ and time‐dependent manner. DMSO increased intracellular and mitochondrial ROS (1.9‐fold and 3.6‐fold respectively after 12 h exposure to 10% DMSO) and downregulated gene expression levels of antioxidant enzymes in a dose‐dependent manner. Tempering ROS through NAC treatment significantly attenuated DMSO‐induced oxidative stress and supported maintenance of cell viability.

**Conclusions:**

This study demonstrated dose‐ and time‐dependent cytotoxic effects of DMSO on human NPC. The addition of NAC to the cryopreservation medium ameliorated cell viability loss by reducing DMSO‐induced oxidative stress in the freeze–thawing cycle. These findings may be useful for future clinical applications of whole cells and cellular products.

## Graphical abstract

N‐acetylcysteine attenuates DMSO induced oxidative stress mediated viability loss in NP cells.

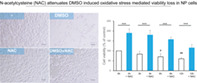

## 1 INTRODUCTION

Low back pain (LBP) is a major cause of disability worldwide and occurs in all age groups, from young to elderly populations.^1^ The health care and work disability costs associated with LBP constitute a tremendous socioeconomic problem.^2^ Although the causes of LBP are multifactorial and often unidentifiable,^3^, ^4^ intervertebral disc (IVD) degeneration is considered the primary etiology of LBP.^5^, ^6^ Aging, mechanical stress, genetics, and other external stimuli can precipitate an imbalance in anabolism and catabolism within the IVDs, provoking a degenerative cascade involving biochemical, biomechanical, and inflammatory changes that can accelerate further degeneration of the disc.^7^, ^8^, ^9^, ^10^, ^11^ Moreover, likely due to the disc's largely avascular nature,^12^ the IVD possesses limited self‐repair capacity.^13^ Progressive IVD degeneration can lead to disc herniation, spinal canal stenosis, and degenerative spondylolisthesis, potentially resulting in lower extremity radicular pain, numbness, muscle weakness, and LBP.^14^, ^15^

Conventional treatments are primarily palliative (e.g., analgesics or physiotherapy) and generally fail to target the underlying pathology. Patients who do not experience pain improvement following conservative interventions, can as a last resort proceed to rather invasive interventions, that is, spinal fusion. However, the efficacy of these surgical treatments remains uncertain.^16^, ^17^, ^18^ The lack of pharmacological or biological therapies targeting IVD degeneration to resolve discogenic LBP and sequential degenerative spinal disease forms a considerable unmet medical need. The field of regenerative medicine has sought to develop new techniques, such as gene therapy,^19^ tissue engineering,^20^, ^21^ and growth factor injection^22^ to tackle the underlying pathogenesis of IVD degeneration in order to alleviate discogenic pain. In particular, cell therapy has shown rapid progression in recent decades^23^ with multiple ongoing clinical trials using a variety of cell types and products, including autologous or allogeneic disc‐derived cells and mesenchymal stem cells.^24^, ^25^, ^26^, ^27^, ^28^ Although an optimal cell type for robust regenerative potential remains to be determined, IVD‐derived nucleus pulposus cells (NPC) are a logical candidate due to their innate adaptability to the harsh IVD environment with the capacity to produce large quantities of IVD‐specific extracellular matrix.^24^, ^28^ We have previously identified Tie2‐positive human nucleus pulposus (NP) progenitor cells with clonal multipotency, optimized a whole‐tissue culture method that enable the expansion and maintenance of Tie2‐positive NPC, and demonstrated their therapeutic potential to treat degenerative disc disease.^29^, ^30^, ^31^, ^32^, ^33^, ^34^, ^35^ Even so, cell transplantation still faces multiple translational and practical hurdles. This includes the need for large quantities of highly potent, safe, and defined cells as a transplantation product to be ready at the time of intervention. This requires the need for reliable cell storage methods; therefore, it is important to develop effective cell cryopreservation techniques that have limited detrimental effects on cell potency. Freeze–thawing cycles are well‐known to affect cell viability and potential, which thereby could reduce therapeutic efficacy.^36^, ^37^ Maintaining cell viability after cryopreservation is a crucial step toward commercialization and off‐the‐shelf (OTS) availability of cell therapy products. The goal for commercialization should be mass production of cell therapy products, ideally producing cell transplantation products for ≥1000 patients from a single donor sample.^35^ In addition, an OTS product that is directly transplanted after thawing without cell culture or removing cryoprotectants is desirable for clinical application, accessibility, and cost reduction. Indeed, some OTS cell products have now been approved for clinical trials.^38^, ^39^, ^40^ Moreover, in mass production of cellular products, when a large volume of cells is cryopreserved at one time, it is inevitable that they are exposed to the cryopreservation medium at harmful temperatures for a certain period of time. This is a critical consideration because dimethyl sulfoxide (DMSO) is the most commonly used cryoprotective agent to prevent intracellular ice formation and has been reported to cause cytotoxicity via elevated oxidative stress and mitochondrial dysfunction.^41^, ^42^, ^43^, ^44^, ^45^ Moreover, multiple studies of other cell types have highlighted the involvement of reactive oxygen species (ROS) as being involved in the detrimental effects of DMSO on cell viability.^45^ While the direct effects of DMSO on human NPC have not been clarified, oxidative stress has been shown to impede NPC proliferation, promote cell senescence, and promote a catabolic phenotype.^46^ Alternatively, DMSO‐containing media have been shown to be detrimental to NPC viability^47^ and differentiation potential.^48^ As such, the purpose of this study was to investigate the effects of DMSO‐induced oxidative stress on human NPC viability and whether the application of antioxidant *N*‐acetylcysteine (NAC; a ROS scavenger)^49^ could potentially mitigate the damage induced through DMSO‐based cryopreservation.

## 2 MATERIALS AND METHODS

### 2.1 Isolation and culture of human NPC

All research procedures described in this study were approved by the Institutional Review Board for Clinical Research, Tokai University (application number 17R‐173). For this study, human IVD tissue samples were collected from 12 patients (mean age ± standard deviation, 20.6 ± 5.0 years) undergoing surgery for lumbar disc herniation at Tokai University Hospital and related facilities (Table 1). All patients provided their informed written consent for the collection and use of surgical waste for research purposes. NPC were isolated and cultured as described previously.^33^, ^35^ Briefly, the collected surgical NP tissue was washed with an excess of 0.9% saline and the tissue was cut into pieces of 3–5 mm in diameter with scissors and scalpels. Culture conditions were employed to mimic the culture conditions of our cell transplantation product in development, following the work of Sako et al.^35^ As such, NP fragments were directly applied to a complete culture medium containing Dulbecco's modified Eagle's medium (DMEM; Gibco, Grand Island, NY, USA) and α‐minimal essential medium (αMEM; Gibco), supplemented with 20% (v/v) fetal bovine serum (FBS; Sigma‐Aldrich, St. Louis, MO, USA) and 1% penicillin/streptomycin (Gibco) and cultured in polystyrene 6‐well plates (IWAKI, Tokyo, Japan). About 0.3 g of NP tissue was seeded per 3 ml culture medium in a single well. Tissue fragments were cultured at 37°C in 5% CO_2_ and 5% O_2_ for 14 days without media replenishment. Subsequently, tissue fragments were collected, centrifuged, and the supernatant was removed. Next, the tissue was resuspended in 10 ml TrypLE Express (Thermo Fisher Scientific, Tokyo, Japan). The suspension was digested with gentle swirling at 37°C for 30 min. Next, the tissue was further digested in a mixture of 10 ml of αMEM supplemented with 10% (v/v) FBS and 0.25 mg/ml collagenase P (Roche, Basel, Switzerland), which was incubated for 2 h at 37°C. After digestion, the suspension was filtered through a 40 μm cell strainer (Corning, NY, USA), centrifuged, and the supernatant was removed. The resulting cells were seeded at a density of 3.0 × 10^4^ cells per well in 100‐mm dishes (Corning) at 37°C in 5% CO_2_ and 5% O_2_ and cultured in medium as previously specified for 7 days without media change. IVD‐derived cells were used following the third passage.

**TABLE 1:** Summary of the patient characteristics of the donor IVD samples. Age, sex, pathology, total weight of IVD tissue collected at the time of surgery, and weight of NP tissue after selection are listed for each sample

Code	Age (year)	Sex	Pathology	IVD Tissue (g)	NP Tissue (g)	Application
A18	18	M	LDH	5.88	3.73	Figure 1A,B, 2B, 4B
T16	16	M	LDH	2.03	2.03	Figure 1A,B, 2D, 3A–E, 4D, 4E, 5A–E
A17	17	M	LDH	4.25	2.99	Figure 1A–J, 4K–N
A14	14	F	LDH	1.74	1.54	Figure 1A–J, 2A–D, 3A–E, 4B, 4D, 4F–N
A18‐2	18	M	LDH	1.74	1.42	Figure 1A,B
A25	25	F	LDH	2.07	1.92	Figure 1A–J, 2B, 2D, 4B, 4D, 4F–I, 4K–N
A16	16	M	LDH	1.81	1.50	Figure 1C–F
A26	26	M	LDH	2.02	1.23	Figure 1C–F, 4F–I
T21	21	M	LDH	5.78	3.83	Figure 1C–F, 4F–I
A21	21	M	LDH	1.75	1.36	Figure 2D, 3A–E, 4C, 4D
T23	23	M	LDH	2.03	1.03	Figure 2B, D, 4B, D, 5A–E
E32	32	F	LDH	4.75	4.74	Figure 2B, 4A, B

Abbreviations: IVD, intervertebral disc; LDH, lumber disc herniation; NP, nucleus pulposus.

### 2.2 Treatment with DMSO and/or NAC

To examine the dose‐dependent effect of DMSO (Nacalai Tesque, Kyoto, Japan), NPC seeded at 1.5 × 10^4^ cells/well in 96‐well plates or 5.0 × 10^4^ cells/well in 6‐well plates were exposed to various concentrations of DMSO ranging from 0.01 to 10% (v/v) prepared in αMEM supplemented with 20% (v/v) FBS for 1 and 24 h. To evaluate the time‐dependent effect of DMSO, NPC were exposed to 5% or 10% DMSO for 3, 6, 12, and 24 h. Furthermore, NPC were treated with a combination of 5% or 10% DMSO and 10 mM NAC (Sigma‐Aldrich) prepared in αMEM supplemented with 20% (v/v) FBS for 3, 6, 12, and 24 h. The concentration of NAC was based on previous work from Seol et al.^50^ and confirmed most effective to retain cell viability (Figure S1). Cells exposed to 0% DMSO were used as an experimental control.

### 2.3 Cell viability assay

Cell viability was determined by luminescence using the CellTiter‐Glo 2.0 Assay (Promega, Madison, WI, USA) according to the manufacturer's instructions. This assay is based on quantification of the presence of ATP, an indicator of metabolically viable cells. Briefly, 1.5 × 10^4^ cells were seeded in each well of a 96‐well plate and DMSO and/or NAC were added to reach the designated final concentration. After incubation for the designated time, 100 μl of CellTiter‐Glo reagents were added in each well, then relative luminescence units were measured using the GloMax 96 microplate luminometer (Promega). Cell viability was expressed as percentage of control values set at 100%.

### 2.4 Apoptosis analysis

Apoptotic and necrotic cells were measured by flow cytometry using APC Annexin V Apoptosis Detection Kit with propidium iodide (PI; BioLegend, San Diego, CA, USA) according to the manufacturer's instructions. Briefly, NPC were seeded at 5.0 × 10^4^ cells/well in 6‐well plates and after treatment with the designated concentrations of DMSO with or without NAC for 24 h, cells were harvested and resuspended in 100 μl of Annexin V binding buffer, supplemented with 5 μl of Annexin V and 5 μl of PI. Samples were incubated in the dark for 15 min at room temperature. The viable (Annexin‐V^−^/PI^−^), early apoptosis (Annexin‐V^+^/PI^−^), late apoptosis (Annexin‐V^+^/PI^+^), and necrosis (Annexin‐V^−^/PI^+^) rates^51^ were analyzed using a BD FACS Canto II flow cytometer (Becton Dickinson, Franklin Lakes, NJ, USA).

### 2.5 Measurement of intracellular and mitochondrial ROS

Cells were seeded at 5.0 × 10^4^ cells/well in 6‐well plates and after treating with the designated concentrations of DMSO with or without NAC for 12 h, NPC were collected, washed twice with phosphate‐buffered saline (PBS), and incubated with 10 μM dihydroethidium (DHE; Invitrogen, Thermo Fisher Scientific, Waltham, MA, USA) or 5 μM MitoSOX Red (Invitrogen) in the dark at 37°C for 30 min. The intracellular and mitochondrial superoxide levels were determined by FACS analysis. The mean fluorescence intensity of DHE and MitoSox was measured using FACS through Phycoerythrin (PE) with excitation at 488 nm and emission at 578 nm. Additionally, NPC were seeded, cultured, and incubated with 10 μM DHE or 5 μM MitoSOX in the dark at 37 °C for 30 min. The plates were washed three times with PBS and then immediately photographed using a fluorescence microscope (BZ‐9000; Keyence Corporation, Osaka, Japan) at settings of ×20 magnification, 100% fluorescence excitation, and a 1.0‐s exposure time.

### 2.6 Real‐time RT‐PCR

NPC seeded at 5.0 × 10^4^ cells/well in 6‐well plates exposed to designated concentrations of DMSO for 24 h, were harvested and total RNA was extracted using ISOGEN II (Nippon Gene, Tokyo, Japan) according to the manufacturer's instructions. Complementary DNA (cDNA) was synthesized from 300 ng of total RNA using High‐Capacity RNA to cDNA Kit (Applied Biosystems, Thermo Fisher Scientific). Based on the TaqMan Gene Expression Assay (Applied Biosystems), gene expression levels were detected using TaqMan Fast Advanced Master Mix and the following primers: TaqMan Gene Expression (human): superoxide dismutase 1 (SOD1; Hs00533490, Applied Biosystems), SOD2 (Hs00167309, Applied Biosystems), SOD3 (Hs04973910, Applied Biosystems), catalase (CAT; Hs00156308, Applied Biosystems), and glutathione peroxidase 1 (GPX1; Hs00829989, Applied Biosystems).

Real‐time PCR was performed using an amplification machine (QuantStudio 3; Applied Biosystems) with stage 1 (95°C for 20 s × 1 cycle) and stage 2 (95°C for 1 s, 60°C for 20 s × 40 cycles). Sample values were normalized by the Ct value for the housekeeping gene 18S (4352930E; Applied Biosystems). The Ct value of control (0% DMSO) was used as a reference, and then relative mRNA expression was calculated using the 2^−ΔΔCt^ method.

### 2.7 Cryopreservation and thawing

The cryopreservation process was performed as described previously.^52^ NPC were trypsinized, washed, and resuspended at 5.0 × 10^5^ cells/ml in serum‐free, animal component‐free cryopreservation medium (CryoStor CS10; BioLife Solutions, Bothell, WA, USA) containing 10% DMSO or a combination of 10% DMSO and 10 mM NAC. The cell suspension (1 ml) was kept on ice for 0, 3, 6, 12 h, after which each cryovial was transferred to a controlled rate freezing container (BICELL; Nippon Freezer, Tokyo, Japan). Upon reaching −80°C, the cryovials were removed from the container and stored in liquid nitrogen. After more than 24 h, the NPC were taken out of the liquid nitrogen and thawed quickly in a water bath at 37°C. Immediately after thawing, cell viability was measured by the CellTiter‐Glo 2.0 Assay using 50 μl of cell suspension. Furthermore, 50 μl of cell suspension was incubated with an equal volume of αMEM (FBS free; to subject cells to a low‐nutrient condition mimicking an IVD environment) or αMEM supplemented with 20% (v/v) FBS and then cell viability was determined after 24 or 72 h. Cell viability was expressed as percentage of control (0 h without NAC) values set at 100%.

### 2.8 Statistical analysis

Statistical analysis was conducted using JMP Pro version 15.0 for Macintosh (SAS Institute, Cary, NC, USA). All data were obtained from at least three independent experiments with at least two technical replicates and presented as mean ± standard deviation (SD). Multiple sets of data were confirmed normally distributed via Shapiro–Wilk test and analyzed via one‐way ANOVA, followed by Dunnett's multiple comparison or Tukey's post hoc test. Student's *t* tests were used in the analysis of two‐group parameters. A *p* value <0.05 was considered statistically significant.

## 3 RESULTS

### 3.1 DMSO decreases cell viability of NPC in a dose‐ and time‐dependent manner

**FIGURE 1:**
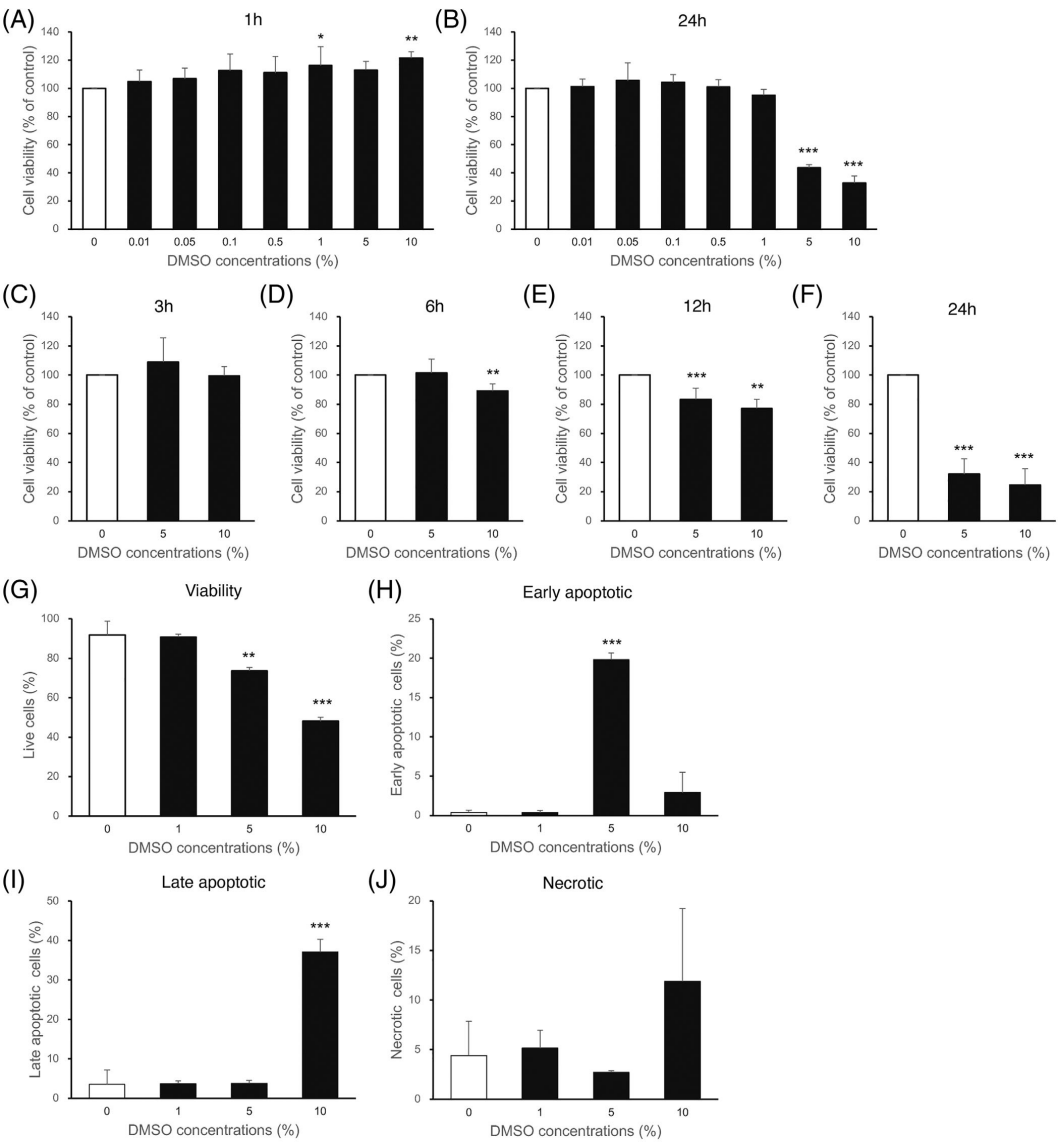
Cytotoxic effects of DMSO on cell viability of NPC. Cell viability was determined by CellTiter‐Glo 2.0 Assay. (A, B) To examine the dose‐dependent effects of DMSO, NPC were exposed to various concentrations of DMSO, widely used as a cryoprotectant and solvent, ranging from 0.01 to 10% (v/v) for 1 and 24 h. (C–F) To evaluate the time‐dependent effects of DMSO, NPC were exposed to 5% or 10% DMSO for 3, 6, 12, and 24 h. (*n* = 6, *p < 0.05, **p < 0.01, ***p < 0.001 vs. 0% DMSO control) (G–J) Rates of cell death were determined for NPC exposed to DMSO for 24 h by Annexin V/ propidium iodide (PI) staining and FACS analysis. (G) Live cells (Annexin‐V^−^/PI^−^) (H) Early apoptotic cells (Annexin‐V^+^/PI^−^) (I) Late apoptotic cells (Annexin‐V^+^/PI^+^) (J) Necrotic cells (Annexin‐V^−^/PI^+^) All data are expressed as mean ± SD. (*n* = 3, **p < 0.01, ***p < 0.001 vs. 0% DMSO control)

To examine the dose‐dependent effects of DMSO, NPC were exposed to various concentrations of DMSO (Figure 1A, B) for 1 and 24 h. Although exposure to DMSO for 1 h did not result in a decrease in cell viability, cell viability was reduced at concentrations above 5% after 24 h exposure to DMSO. To evaluate the time‐dependent effects of DMSO, NPC were exposed to 5% or 10% DMSO for 3, 6, 12, and 24 h (Figure 1C–F). Exposure to DMSO within 3 h did not cause a decrease in cell viability, whereas exposure to DMSO for more than 6 h significantly decreased cell viability in a time‐dependent manner (Figure 1D–F), with a loss of 57% and 68% after 24 h exposure to 5% and 10% DMSO, respectively. These results demonstrate the dose‐ and time‐dependent cytotoxic effects of DMSO on NPC.

### 3.2 DMSO induces apoptosis and necrosis of NPC

DMSO‐induced cell viability loss was related to the induction of apoptosis or necrosis through Annexin V/PI staining (Figure 1G–J). Again, exposure to DMSO concentrations of 5% and 10% showed a significant reduction in viable populations (Figure 1G). Interestingly, exposure to 5% DMSO only significantly increased early apoptosis populations (20%) (Figure 1H), while exposure to 10% DMSO significantly increased late apoptosis populations (37%) and showed higher rates of necrosis (Figure 1I–J).

### 3.3 DMSO induces intracellular and mitochondrial ROS of NPC

**FIGURE 2:**
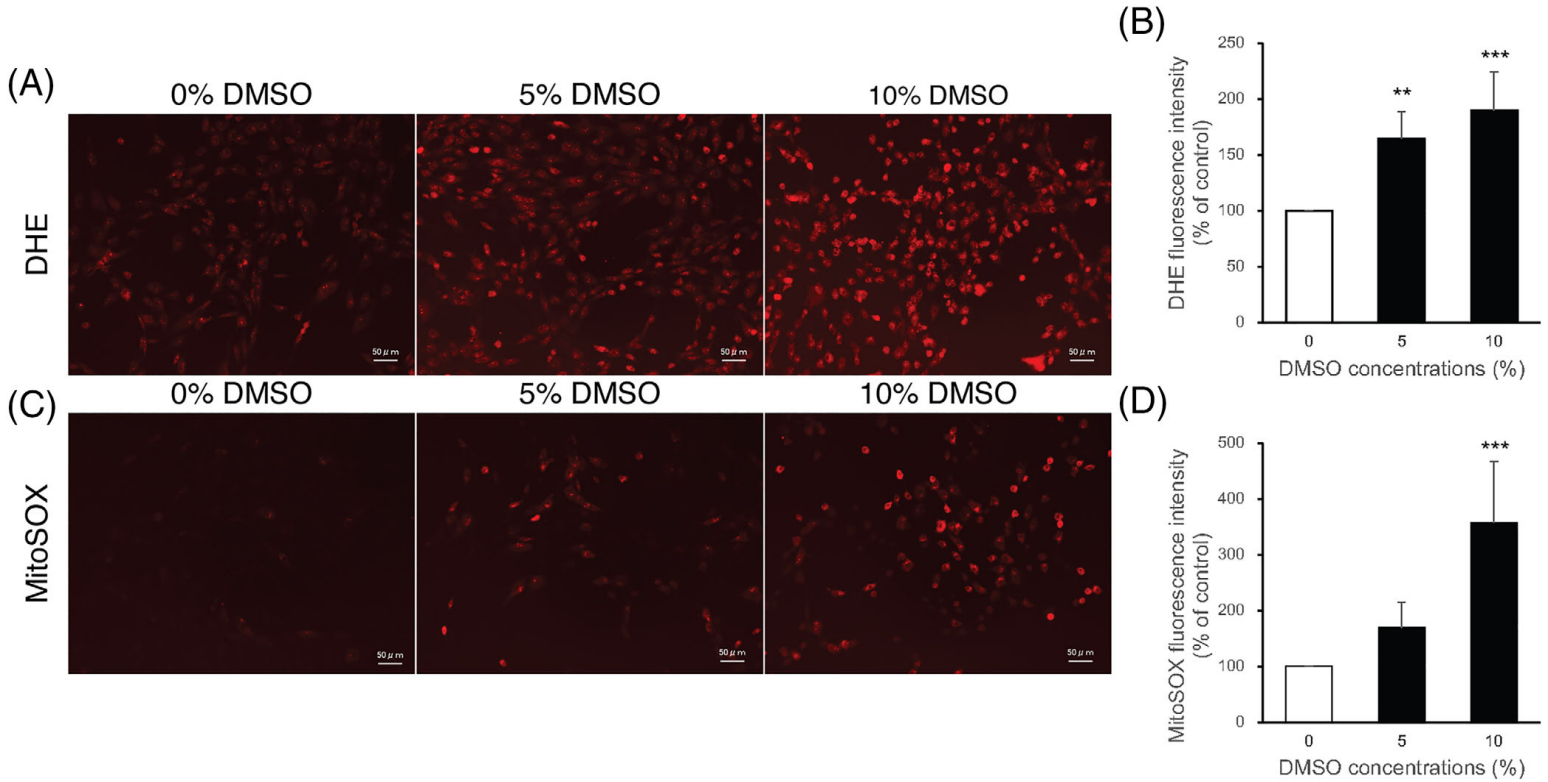
Effects of DMSO on reactive oxygen species (ROS) production of NPC. The intracellular and mitochondrial superoxide levels in DMSO‐treated NPC were determined by dihydroethidium (DHE) and MitoSOX Red staining, respectively. (A) Representative fluorescence microscopic images of DHE staining. (Scale bars = 50 μm) (B) Quantitative analysis of mean DHE fluorescence intensity. (C) Representative fluorescence microscopic images of MitoSOX staining. (Scale bars = 50 μm) (D) Quantitative analysis of mean MitoSOX fluorescence intensity. All data are expressed as mean ± SD. (*n* = 5, **p < 0.01, ***p < 0.001 vs. 0% DMSO control)

Oxidative stress caused by excessive ROS generation is one of the principal factors leading to apoptosis. The generation of intracellular and mitochondrial ROS in DMSO‐treated NPC was assessed by FACS and fluorescence microscopy using DHE and MitoSOX Red staining. Exposure to DMSO significantly increased oxidized DHE (red) intensity, indicating an increase in intracellular ROS levels (Figure 2A, B). This increase occurred in a dose‐dependent manner. Furthermore, MitoSOX staining highlighted that exposure to 5% DMSO increased and 10% DMSO significantly increased mitochondrial ROS levels (Figure 2C, D).

### 3.4 DMSO downregulates gene expression of antioxidant enzymes in NPC

**FIGURE 3:**
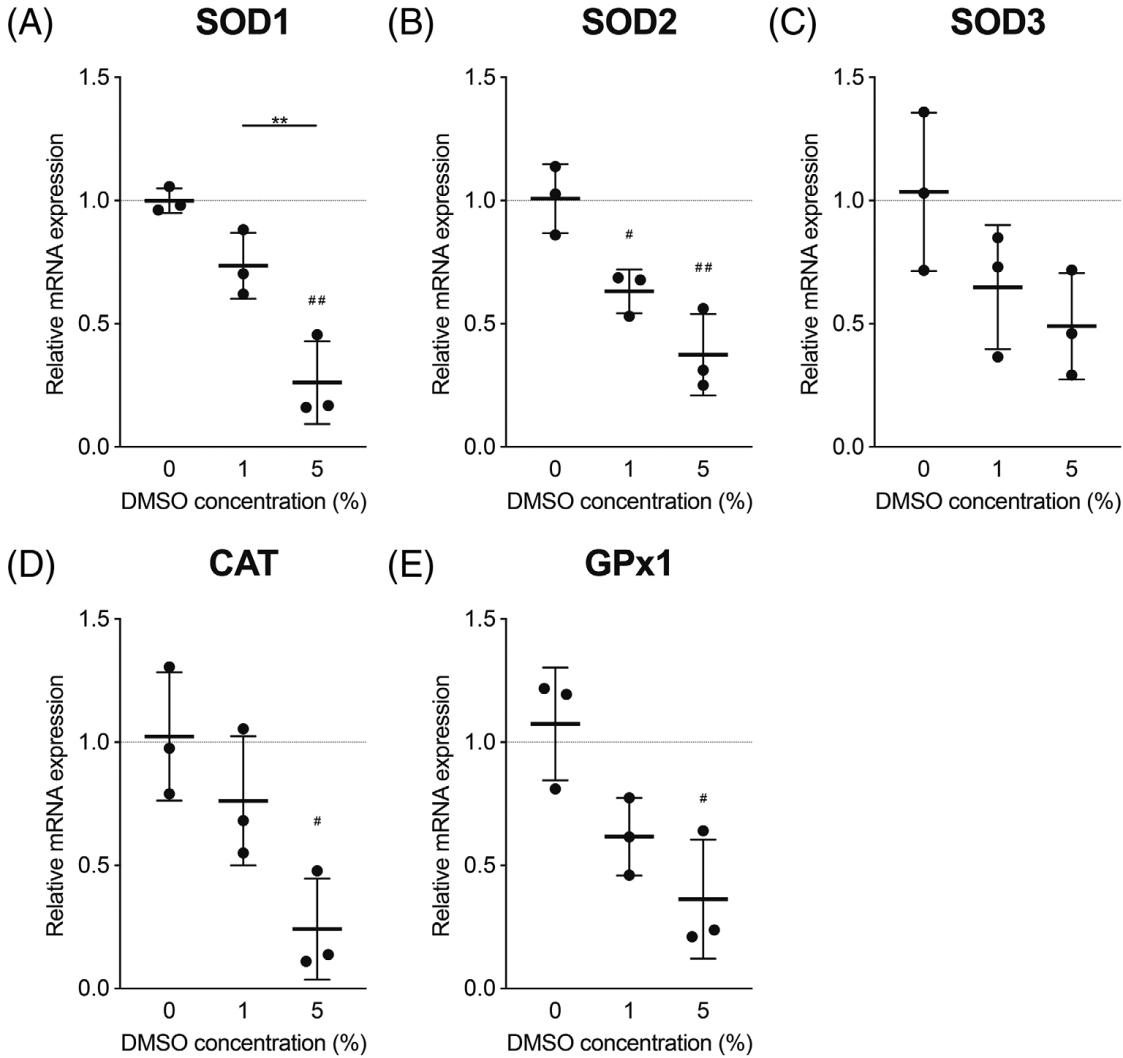
Effects of DMSO on gene expression levels of antioxidant enzymes in NPC exposed to DMSO. Relative mRNA expression of (A) SOD1, (B) SOD2, (C) SOD3, (D) CAT, and (E) GPX1. All data are expressed as mean ± SD. (*n* = 3, ^#^ *p* < 0.05, ^##^ *p* < 0.01 vs. 0% DMSO control, **p < 0.01 vs. 5% DMSO)

To investigate whether DMSO affects the expression pattern of antioxidant enzymes, the gene expression of the major antioxidant enzymes in DMSO‐treated NPC was evaluated. Interestingly, exposure to DMSO in a dose‐dependent manner significantly downregulated gene expression of antioxidant enzymes SOD1 and SOD2 and showed a clear trend of reduction for SOD3 mRNA level (Figure 3A–C). Moreover, antioxidant enzymes, CAT and GPx1 similarly showed a reduction in expression levels with increasing concentration of DMSO, resulting in a significant reduction in NPC exposed to 5% DMSO (Figure 3D–E).

### 3.5 NAC attenuates DMSO‐induced cell viability loss and cell death of NPC

**FIGURE 4:**
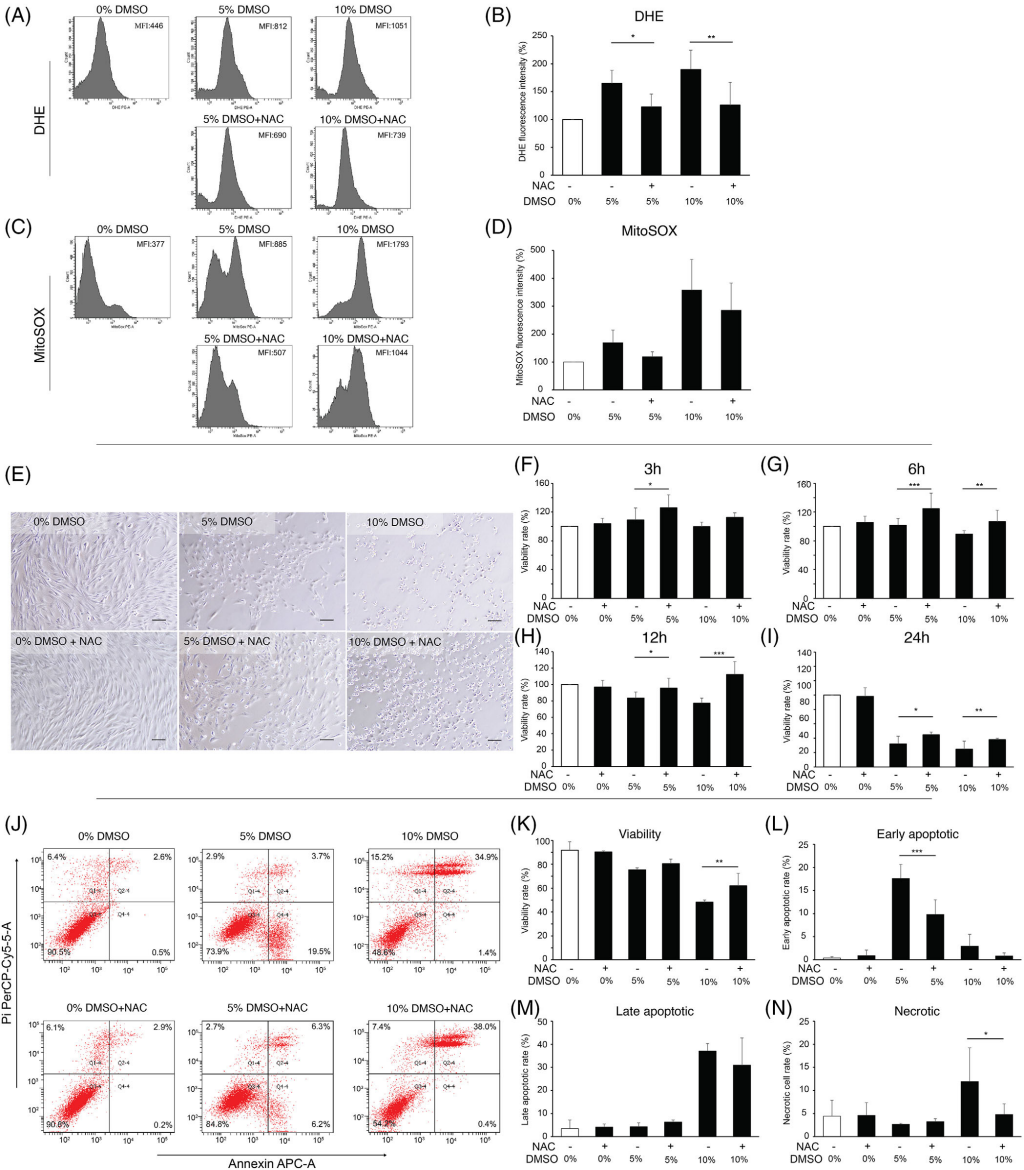
Cytoprotective effects of N‐acetylcysteine (NAC) against DMSO‐induced cell viability loss in NPC. (A) Representative flow cytometry graphs of DHE staining. (B) Quantitative analysis of mean DHE fluorescence intensity. (*n* = 5) (C) Representative flow cytometry graphs of MitoSOX staining. (D) Quantitative analysis of mean MitoSOX fluorescence intensity. (*n* = 5) (E) Representative phase contrast microscopic images of cultured NPC. (Scale bars = 100 μm) (F–I) The effects of NAC against DMSO‐induced cell viability loss with exposure for 3, 6, 12, and 24 h respectively (*n* = 4). (J) Representative flow cytometry plots of Annexin V/PI staining. (K–N) The effects of NAC against DMSO‐induced cell death, depicting the rates of (K) viable cells, (L) early apoptotic cells, (M) late apoptotic cells, and (N) necrotic cells. (*n* = 3) All data are expressed as mean ± SD. (*p < 0.05, **p < 0.01, ***p < 0.001, NAC‐treated vs. corresponding untreated cells)

To examine whether DMSO‐induced cell viability loss and cell death could be prevented by inhibiting ROS generation, DMSO‐treated NPC were subjected to 10 mM NAC.^50^ The addition of NAC significantly decreased DMSO‐induced intracellular ROS generation (Figure 4A, B) and tended to decrease mitochondrial intracellular ROS generation (Figure 4C, D). Furthermore, NAC significantly attenuated DMSO‐induced cell viability loss at every point in time for both 5% and 10% DMSO concentration (Figure 4E–I). Strikingly, NAC was able to maintain viability levels similar to those of nontreated NPC for up to 12 h (Figure 4H). In addition, NAC significantly attenuated DMSO‐induced cell apoptosis and necrosis (Figure 4J–N). Notably, NAC inhibited DMSO‐induced apoptotic morphologic features, including cell rounding, cell shrinkage, and cell detachment from the plates (Figure 4E). Taken together, these results suggest that ROS plays an important role in DMSO‐induced cell viability loss and apoptosis.

### 3.6 NAC ameliorates DMSO‐induced cell viability loss during the freeze–thawing cycle

**FIGURE 5:**
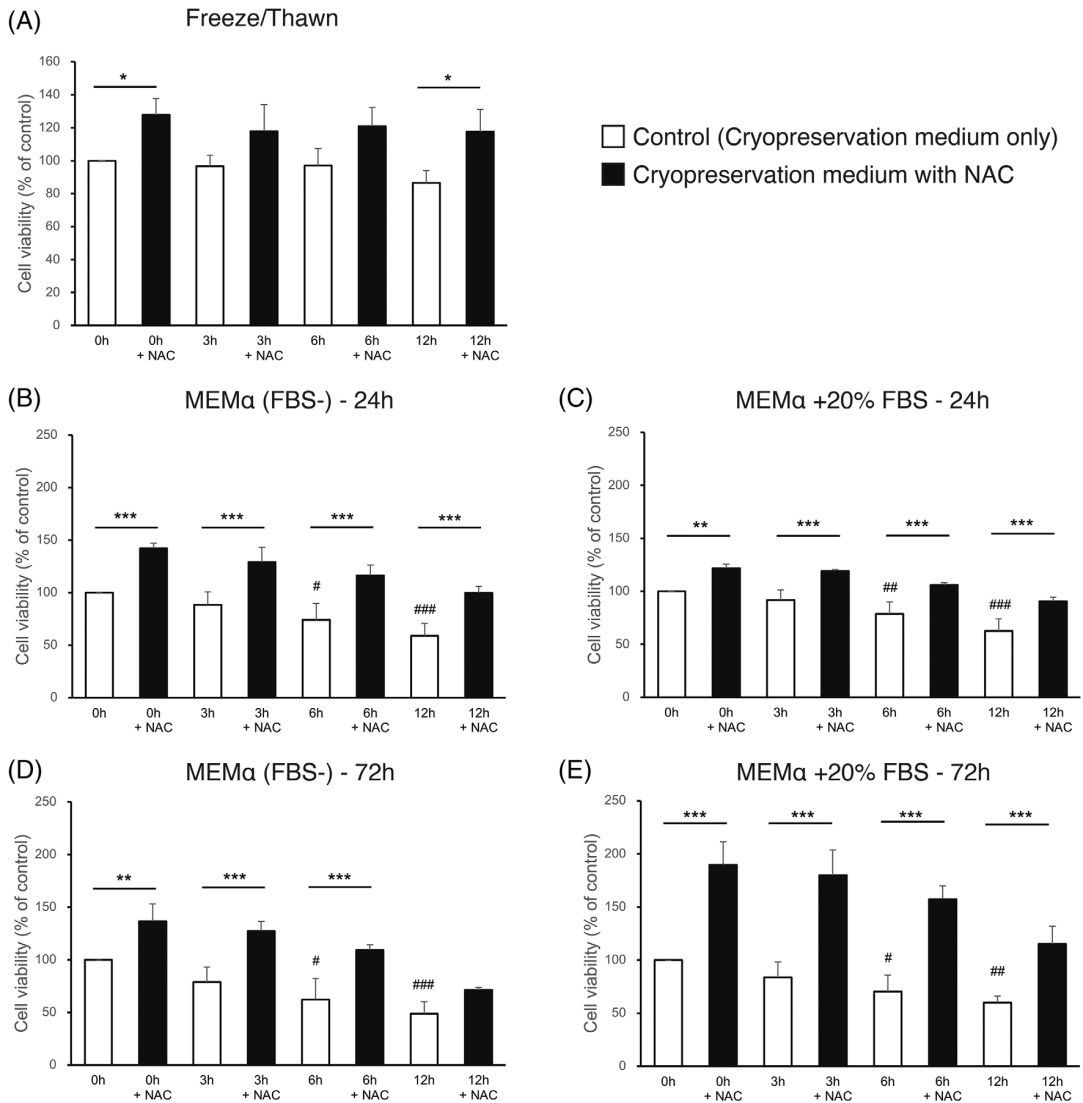
Cytoprotective effects of N‐acetylcysteine (NAC) against DMSO‐induced cell viability loss of NPC following freeze‐thawing processes. To examine the effects of NAC addition and pre‐freeze exposure times to cryopreservation medium containing 10% DMSO on cell viability following a freeze‐thawing processes, cell viability was determined (A) immediately after thawing, or after 24 h of incubation with an equal volume of (B) αMEM (FBS free) or (C) αMEM supplemented with 20% (v/v) FBS. Also, (D) cell viability after 72 h of incubation with an equal volume of αMEM (FBS free) or (E) with an equal volume of αMEM supplemented with 20% (v/v) FBS, was determined. All data are expressed as mean ± SD. (*n* = 4, ^#^ *p* < 0.05, ^##^ *p* < 0.01, ^###^ *p* < 0.001 vs. 0 h without NAC control, *p < 0.05, **p < 0.01, ***p < 0.001, NAC‐treated vs. corresponding untreated cells)

Finally, the effects on cell viability of NAC addition to cryopreservation medium containing 10% DMSO during the freeze–thawing cycle were examined. Cell viability appeared not to be affected by prefreeze exposure time to cryopreservation medium. However, the addition of NAC significantly increased cell viability for each condition (Figure 5A). Furthermore, cell viability evaluated after 24 and 72 h of incubation with an equal volume of culture medium, aiming to mimic cell transplantation products, revealed that exposure to cryopreservation medium for 3 h prior to freezing did not cause a significant decrease in cell viability, whereas exposure for more than 6 h significantly decreased cell viability at both 24 and 72 h after thawing (Figure 5B–E). Notably, the addition of NAC significantly increased cell viability at all time points (Figure 5B–E), and in some cases even doubled viability rates (Figure 5E).

## 4 DISCUSSION

This study highlights the association of intracellular and mitochondrial ROS with the cytotoxic effects observed in human NPC when exposed to DMSO. Moreover, the DMSO‐induced cell viability loss could be ameliorated through supplementation of NAC to the cryopreservation medium, offering a potentially simple and cost‐effective method for enhancing OTS cell product potency.

Although high concentrations of DMSO (≥ 10% v/v) have previously been shown to induce cytotoxicity through plasma membrane pore formation,^41^, ^53^, ^54^, ^55^ the dose‐ and time‐specific effects of DMSO on human NPC and the exact mechanisms involved in DMSO‐induced cytotoxicity on NPC remain unclear. In this study, exposure to DMSO at concentrations ≤1% for 24 h did not significantly affect the cell viability of NPC, whereas exposure to 5%–10% DMSO, a concentration commonly contained in cryopreservation media, for more than 6 h caused cell viability loss and cell death in a dose‐ and time‐dependent manner.

There is accumulating evidence that ROS generation caused by mitochondrial dysfunction plays a central role in initiating apoptosis.^56^, ^57^, ^58^ Moreover, oxidative stress caused by excessive ROS generation is one of the major causes leading to cell death.^59^, ^60^ Previous studies have shown that DMSO induces apoptosis via elevated ROS and mitochondrial dysfunction. In adipocytes, for example, DMSO at concentrations ≥1% for 1 h reduced cell viability and accelerated cellular apoptosis by increasing mitochondrial membrane potential and ROS.^42^ Elsewhere, in cardiomyoblasts and in breast cancer cells, 3.7% DMSO exposure for 6 days induced apoptosis driven by mitochondrial dysfunction and oxidative stress.^44^ Finally, exposure of astrocytes to 5% DMSO for 24 h caused cell viability loss and mitochondrial integrity disruption, membrane potential impairment, ROS production, and subsequent cytochrome C release and caspase‐3 activation.^45^ Consistent with these previous reports, we demonstrated that DMSO increases intracellular and mitochondrial ROS in NPC in a dose‐dependent manner. Interestingly, DMSO downregulated the gene expression of major antioxidant enzymes in a dose‐dependent manner. This suggests that DMSO induces excessive ROS production, which depletes intracellular antioxidant defense systems, and the resulting redox imbalance induces cell death. Furthermore, inhibiting ROS generation by NAC treatment significantly attenuated DMSO‐induced cell death. Taken together, these results suggest that ROS plays a pivotal role in DMSO‐induced cell viability loss.

NAC is a well‐established drug that was originally employed as a medical agent against acetaminophen overdose or as a mucolytic agent in bronchitis and pneumatic diseases. Moreover, NAC is well established as an antioxidant (precursor), although the primary mechanism involved in its antioxidative activities remains contested.^61^ NAC's application is being examined in a wide range of different diseases and for different applications.^62^ In our study, the rationale and benefits of NAC addition to the cryopreservation medium appears to be twofold. First, the addition of NAC may have increased cell viability after the freeze–thawing cycle by inhibiting DMSO‐induced ROS generation or reducing oxidative stress associated with the freeze–thawing processes. Previous studies have demonstrated that the changes in temperature and osmolarity during the freeze–thawing process lead to increased ROS, mitochondrial damage, and cell apoptosis.^36^, ^37^, ^63^, ^64^ Thus, our results suggest that NAC could protect cells from oxidative stress by acting as a ROS scavenger. Second, direct administration of NAC, for example as part of an OTS cell therapy product, into the IVDs is expected to have some beneficial effects on IVD homeostasis. For example, Suzuki et al.^65^ showed that oxidative stress contributes to the progression of IVD degeneration and oral administration of NAC was able to moderate induced IVD degeneration in a rat model. Furthermore, in scaffold and organ cultures of rat NPC, NAC inhibited ROS generation, cell senescence, and decreased matrix synthesis promoted by high‐magnitude compression.^66^ Based on the above, the addition of NAC to cryopreservation medium may be useful in consideration of direct transplantation of OTS cell therapy products into the IVDs.

Cryopreserving cells provides many benefits for clinical use and commercialization, such as long‐term storage, OTS usability, and the ability to complete safety and functional testing of the cells prior to human dosing.^25^ We have previously demonstrated that cell transplantation of human discogenic cells directly from their cryopreserved state is an effective and safe strategy, and has the potential to provide an OTS cell therapy product for the treatment of degenerative disc disease.^34^, ^67^ As such, the findings of this study may be a promising discovery for future clinical applications of cell product development toward IVD regeneration.

Our study has several limitations. First, the biological and functional characteristics of NPC after the freeze–thawing cycle, such as matrix synthesis capacity have not been evaluated. Second, our experiments were only executed through in vitro experiments. As such, the effect of DMSO and NAC on NPC in vivo or as cell therapy products was not evaluated. Future studies will need to evaluate their effect in vivo through transplantation of NPC loaded into DMSO and/or NAC‐containing media.

In summary, this study showed the dose‐ and time‐dependent cytotoxic effects of DMSO on human NPC. Moreover, intracellular ROS played a critical role in DMSO‐induced cell viability loss. The addition of NAC to cryopreservation medium ameliorated cell viability loss by reducing DMSO‐induced oxidative stress before and after the freeze–thawing cycle. These results may help the development of cell therapy products for IVD regeneration.

## AUTHOR CONTRIBUTIONS

Shota Tamagawa participated in the design of the study, performed experiments, analyzed data, and wrote the paper. Daisuke Sakai participated in the design and coordination. Jordy Schol analyzed data and wrote the paper. Kosuke Sako, Yoshihiko Nakamura, Erika Matsushita, Takayuki Warita, Soma Hazuki participated in the design and performed experiments. Hidetoshi Nojiri, Masato Sato, Muneaki Ishijima, Masahiko Watanabe performed critical revision for intellectual content. All authors read and approved the final manuscript.

## CONFLICT OF INTEREST

Author DS is an unpaid scientific advisor for TUNZ Pharma (Osaka, Japan). TW and SH are paid employees of TUNZ Pharma (Osaka, Japan).

## Supporting information


**Figure S1** Cytoprotective effects of N‐acetylcysteine (NAC) against DMSO‐induced cell viability loss of NPC. (A–C) To examine the dose‐dependent effects of NAC against DMSO‐induced cell viability loss with exposure for 24 h, NPC were exposed to various concentrations of NAC, ranging from 0.01 to 10 mM. All data are expressed as mean ± SD. (*n* = 3, *p < 0.05, vs. control without NAC)
