# Supplementary material for: N‐acetylcysteine attenuates oxidative stress‐mediated cell viability loss induced by dimethyl sulfoxide in cryopreservation of human nucleus pulposus cells: A potential solution for mass production
Source: JOR Spine. 2022 Oct 1;5(4):e1223. doi: 10.1002/jsp2.1223 (PMC9799083; doi:10.1002/jsp2.1223)
Supplement: Supplementary file 1 — Figure S1 Cytoprotective effects of N‐acetylcysteine (NAC) against DMSO‐induced cell viability loss of NPC. (A–C) To examine the dose‐dependent effects of NAC against DMSO‐induced cell viability loss with exposure for 24 h, NPC were exposed to various concentrations of NAC, ranging from 0.01 to 10 mM. All data are expressed as mean ± SD. (n = 3, *p < 0.05, vs. control without NAC) [file JSP2-5-e1223-s001.docx]

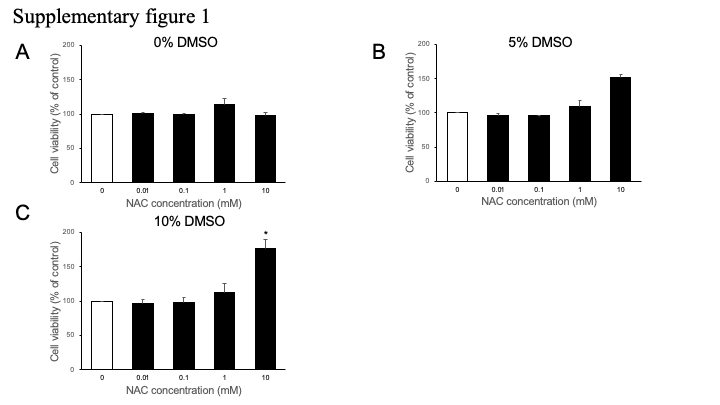


Supplementary figure 1. Cytoprotective effects of N-acetylcysteine (NAC) against DMSO-induced cell viability loss of NPC. (A-C) To examine the dose-dependent effects of NAC against DMSO-induced cell viability loss with exposure for 24 h, NPC were exposed to various concentrations of NAC, ranging from 0.01 to 10 mM. All data are expressed as mean ± SD. (n = 3, *P < 0.05, versus control without NAC)
